# HMGB1 Is Associated with Atherosclerotic Plaque Composition and Burden in Patients with Stable Coronary Artery Disease

**DOI:** 10.1371/journal.pone.0052081

**Published:** 2012-12-17

**Authors:** Martin Andrassy, H. Christian Volz, Alena Schuessler, Gitsios Gitsioudis, Nina Hofmann, Danai Laohachewin, Alexandra R. Wienbrandt, Ziya Kaya, Angelika Bierhaus, Evangelos Giannitsis, Hugo A. Katus, Grigorios Korosoglou

**Affiliations:** 1 Department of Cardiology, University of Heidelberg, Heidelberg, Germany; 2 Department of Internal Medicine III and Clinical Chemistry, University of Heidelberg, Heidelberg, Germany; University of Freiburg, Germany

## Abstract

**Objectives:**

The role of inflammation in atherosclerosis is widely appreciated. High mobility group box 1 (HMGB1), an injury-associated molecular pattern molecule acting as a mediator of inflammation, has recently been implicated in the development of atherosclerosis. In this study, we sought to investigate the association of plasma HMGB1 with coronary plaque composition in patients with suspected or known coronary artery disease (CAD).

**Design:**

HMGB1, high sensitive troponin T (hsTnT) and high sensitive C-reactive protein (hsCRP) were determined in 152 consecutive patients with suspected or known stable CAD who underwent clinically indicated 256-slice coronary computed tomography angiography (CCTA). Using CCTA, we assessed 1) coronary calcification, 2) non-calcified plaque burden and 3) the presence of vascular remodeling in areas of non-calcified plaques.

**Results:**

Using univariate analysis, hsCRP, hsTnT and HMGB1 as well as age, and atherogenic risk factors were associated with non-calcified plaque burden (r = 0.21, p = 0.009; r = 0.48, p<0.001 and r = 0.34, p<0.001, respectively). By multivariate analysis, hsTnT and HMGB1 remained independent predictors of the non-calcified plaque burden (r = 0.48, p<0.01 and r = 0.34, p<0.001, respectively), whereas a non-significant trend was noticed for hs-CRP (r = 0.21, p = 0.07). By combining hsTnT and HMGB1, a high positive predictive value for the presence of non-calcified and remodeled plaque (96% and 77%, respectively) was noted in patients within the upper tertiles for both biomarkers, which surpassed the positive predictive value of each marker separately.

**Conclusions:**

In addition to hs-TnT, a well-established cardiovascular risk marker, HMGB1 is independently associated with non-calcified plaque burden in patients with stable CAD, while the predictive value of hs-CRP is lower. Complementary value was observed for hs-TnT and HMGB1 for the prediction of complex coronary plaque.

## Introduction

Despite recent advances in medical and interventional treatment strategies coronary artery disease (CAD) remains the leading cause of myocardial infarction and sudden cardiac death in industrialized countries[1], [2]. In patients with acute myocardial infarction, the rupture of coronary plaques with initiation of thrombus formation and subsequent embolization of atherosclerotic debris result in myocardial cell necrosis. However, atherosclerotic plaque development occurs ‘silently’ over several decades before the clinical manifestation of acute coronary syndromes[3], [4].

Currently, non-invasive imaging of coronary vessels is feasible using coronary computed tomography angiography (CCTA), which allows the evaluation of the coronary vessel wall, in addition to the assessment of coronary lumen narrowing[5]. Such characterization of coronary atherosclerotic lesions was shown to have incremental value for the assessment of cardiovascular risk and prediction of future cardiac events compared to clinical parameters and coronary calcification[6]–[8].

Biochemical markers on the other hand, can be easily acquired and can help understanding the underlying pathophysiology of coronary atherosclerosis development and progression. In this regard, we recently demonstrated that high mobility group box 1 (HMGB1, also known as amphoterin) protein is a critical mediator of in acute experimental ischemic injury[9] and predicts outcome after myocardial infarction[10]. In addition, we and others recently reported that high sensitive troponin T (hs-TnT), a well established marker of cardiovascular risk, is associated with composition of atherosclerotic plaque on CCTA images [11], [12].

In the present study we sought to investigate the association of plasma HMBG1 with coronary calcification and with non-calcified plaque composition in patients with suspected or known stable CAD. The acquired results were compared to (i) clinical variables, (ii) hs-TnT, and (iii) high sensitive C-reactive protein (hs-CRP), a marker of low-grade systemic inflammation.

## Materials and Methods

### Study Population

The study population consisted of 152 consecutive patients scheduled to undergo clinically indicated cardiac CTA for suspected or known CAD. Exclusion criteria were non-sinus rhythm, acute coronary syndromes, moderate or severe valvular disease, elevated serum creatinine (>1.5 mg/dl) and history or ECG signs of previous myocardial infarction. All patients underwent 2D-echocardiography before enrolment and patients with impaired systolic ejection fraction (<55%) or presence of regional wall motion abnormalities were also excluded from analysis.

Traditional risk factors for CAD, including arterial hypertension (blood pressure≥140/90 mmHg or antihypertensive therapy), hyperlipidemia (low-density lipoprotein cholesterol (LDL-C)≥3.5 mmol/L or statin therapy), current or prior smoking, diabetes mellitus, and a family history of CAD were recorded at the time of the CT scans.

The CTA protocol included the intravenous administration of incremental doses of 2.5 mg of metoprolol (range 2.5–25.0 mg), (Lopresor®, Novartis, Pharma GmbH) starting 10–20 min before CTA in patients with heart rates ≥65beats/min. If the heart rate remained ≥65beats/min despite the administration of metoprolol, a retrospective scan was performed. If the heart rate decreased to <65beats/min, prospective CTA scans were acquired. Furthermore, sublingual glyceryl nitrate was administrated before CTA for coronary vasodilatation in all patients. All procedures complied with the Declaration of Helsinki, were approved by our local ethic committee and all patients gave written informed consent.

### 256-slice CT Scanning Technique

CT scans were performed using a 256-slice Brilliance iCT scanner (Philips Healthcare) that features a gantry rotation time of 270 ms, resulting in a temporal resolution of 36–135 ms, depending on the heart rate of the patient and the reconstruction mode, and an isotropic sub-millimeter spatial resolution.

- **Coronary calcium scoring**. For the assessment of coronary calcification prospective ECG-gated non-contrast scans were performed at 75% of the cardiac cycle, and using 120 kV tube voltage and 364 mA tube current, and resultant images with a 3 mm slice thickness were used for the calculation of the Agatston score.
- **CT Angiography (CTA) and estimation of the radiation dosage**. For CTA a bolus of 80 ml of contrast agent (Ultravist 370, Bayer Healthcare) was injected intravenously (6 ml/s). As soon as the signal in the descending aorta reached a predefined threshold of 100 HU, the scan started automatically and the entire volume of the heart was acquired during one breath-hold in 4–7 s with simultaneous ECG recording. The detector collimation was 2×128×0.625 mm, with 256 overlapping slices of 0.625 mm thickness and dynamic z-focal spot. The tube voltage was 120 kV and the gantry rotation time was 0.27s. A current of 800–1050 mAs (depending on patient habitus) was used for retrospective and a current of 200 mAs for prospective acquisitions. With retrospective acquisitions reconstructions were routinely performed at 40%, 70%, 75% and 80% of the cardiac cycle. With prospective acquisitions reconstructions were available at 75% of the cardiac cycle. The effective dose was calculated for all CTA scans, based on the dose length product (DLP) and an organ weighting factor for the chest as the investigated anatomic region (k = 0.014 mSv×(mGy×cm)-1) averaged between male and female models[13].

### Assessment of Plaque Volume and Composition

CTA data sets were anonymized and were analyzed in random order using commercially available software (Philips Extended Brilliance Workspace 4.0). The composition of atherosclerotic plaques was performed using the Plaque SW version 4.0.2, as described previously [5]. Briefly, for each coronary artery the vessel lumen and wall were automatically registered, and after the identification of each lesion the boundaries were manually edited if necessary. Subsequently, the identified plaques were marked, and the validity of the proposed lesion areas was evaluated in adjacent cross-sectional multi-planar reconstructed images of the coronaries. Care was taken to correctly discriminate between iodinated blood (300–600 HU) and calcified plaque, and Gaussian algorithms were used to distinguish between components of low to intermediate attenuation (0 to 150 HU) and calcified plaque components with higher attenuation values. By this model, components with different signal intensity within the plaque are separated, using a mixture Gaussian model, into a linear combination which includes 1–3 Gaussians curves. For each coronary lesion the following parameters were assessed:

1. Non-calcified plaque volume for each individual lesion and per patient, by summing up the individual volumes of low-attenuation or mixed plaques in all coronaries,
2. Plaque composition by percentage of calcified content. According to the calcium content, plaques were classified into (i) low-attenuation (calcium content<20%), (ii) mixed (calcium content between 20%–80%) and (iii) calcified (calcium content>80%). Low-attenuation and mixed plaques (with non-calcified content≥20%) are expected to contain substantial amount of lipid cores or fibrotic tissues[7] apart from calcified tissue, and will be addressed to as ‘non-calcified’ plaques throughout our manuscript.
3. Vascular remodeling, which was defined as a change in the vessel diameter at the plaque site in comparison to the reference segment proximal to the lesion (reference diameter). Quantification of the vessel diameters in longitudinal reconstructions, was used to assess the remodeling index (lesion diameter/reference diameter), which was considered positive when the diameter at the plaque site was ≥10% larger than that measured in the reference segment[7], [11].

### Measurement of High Sensitive Troponin T (hsTnT), High Sensitive CRP (hsCRP) and HMBG1

Blood samples were collected from all patients within 2 hours before the CTA scans, centrifuged and serum aliquots were stored at −80°C until analysis. For hs-TnT quantification an ELECSYS 2010 automated analyzer was used (Roche Diagnostics, Mannheim, Germany). The diagnostic range of this assay is 3 to 10000 pg/ml with an inter-assay coefficient of variation of 8% at 10 pg/ml, and 2.5% at 100 pg/ml. The intra-assay coefficient of variation is 5% at 10 pg/ml and 1% at 100 pg/ml. Hereby, based on a healthy reference population, an upper reference limit of 14 pg/ml (99th percentile for TnT) is recommended. HsCRP was quantified by nephelometry, utilizing polystyrene bead- coupled antibodies (Siemens Healthcare Diagnostics, Eschborn, Germany). HMGB1 measurement was performed using ELISA (Shino-Test Corp., Kanagawa, Japan, distributed by IBL, Hamburg, Germany) according to the manufacturer’s instructions[14] with an intra- and inter-assay coefficient of variation of <10%.

### Follow-up Data

Personnel unaware of the stress results contacted each subject or an immediate family member and the date of this contact was used for calculating the follow-up time duration. Outcome data were collected from a standardized questionnaire and determined from patient interviews at the outpatient clinic or by telephone interviews. Reported clinical events were confirmed by review of the corresponding medical records in our electronic Hospital Information Systems (HIS), contact with the general practitioner, referring cardiologist or the treating hospital. Death, myocardial infarction and clinically indicated coronary revascularization procedures by PCI or CABG were defined as major cardiac adverse events (MACE) during the follow-up period.

### Statistical Analysis

Analysis was performed using commercially available software MedCalc9.3 (MedCalc software, Mariakerke, Belgium) and data are presented as mean±one standard deviation. The relation between Agatston score and total non-calcified plaque volume with hsTnT, HsCRP and HMGB1 was assessed using linear regression analysis. Differences in hsTnT and hsCRP levels by plaque composition and with or without vascular remodeling were assessed using ANOVA with Bonferroni’s adjustment for multiple comparisons. Furthermore, CTA findings for calcium scoring and plaque composition were analyzed by patient tertiles based on the corresponding hsTnT and HMBG1 values. Uni- and multivariate logistic regression analysis was used to estimate the ability clinical variables and biochemical markers to predict non-calcified plaque burden, plaque composition and clinical outcome. Linear regression analysis was used to investigate the relation between calcium scoring and coronary plaque burden with biochemical markers. Intra- and inter-observer variability for quantification of 1) non-calcified plaque volume, 2) coronary calcium with non-contrast scans and 3) plaque subtype categorization were calculated by repeated analysis of 40 randomly selected cases. Differences were considered statistically significant at p<0.05.

## Results

### Clinical Characteristics and CCTA Results

Clinical, demographic and biochemical data of our 152 patients are presented in Table 1. Overall, an intermediate risk profile was noted with a mean age of 64±10yrs, 87 (57%) male patients and a mean score of 2.5±1.1 atherogenic risk factors. The majority of our patients did not have obstructive CAD (42 (28%) without plaque or stenosis and 75 (49%) with <50% stenosis), whereas 35 (23%) had obstructive CAD (≥50% diameter stenosis), including 18 (12%) with single- and 17 (11%) with multi-vessel CAD.

**Table 1:** Demographic and cardiac CT data.

Parameters	Patients (n = 152)
	***Demographics***
*Age (yrs)*	64±10
*Male sex*	87 (57%)
	***Coronary risk factors***
*Arterial hypertension*	121 (80%)
*Hypercholesterolemia*	87 (57%)
*Diabetes mellitus*	14 (9%)
*Family history of coronary artery disease*	70 (46%)
*Smoking*	64 (42%)
*Total number of risk factors (0–5)*	2.5±1.1
	***Cardiac medications***
*Aspirin (100* *mg/day) or clopidogrel* *(75* *mg/day)*	84 (55%)
*β-blockers*	75 (49%)
*ACE inhibitors or angiotensin receptor* *blockers*	42 (28%)
*Statins*	59 (39%)
*Nitrates*	5 (3%)
	***Calcium scoring and CTA data***
*Heart rate(1/min)*	62±9
*Metoprolol administration I.V. (mg)*	6.0±5.8
*Calcium Scoring (Agatston units)*	148±193
*No plaques or stenosis*	42 (28%)
*Diameter stenosis <50%*	75 (49%)
*Single vessel CAD*	18 (12%)
*Multi vessel CAD*	17 (11%)
	***Biochemical markers***
*Hs-CRP (mg/dl)*	6.1±2.3
*Hs-TnT (pg/ml)*	10.7±6.1
*Hmbg1 (ng/ml)*	2.8±4.7

Data presented as number of patients or as mean±standard deviation.

### Image Quality and Radiation Exposure

Diagnostic image quality was achieved in 2257 of 2280 available coronary segments (99.0%). In 114 of 152 patients (75%) prospective CTA scans were acquired (mean dose of 2.8±0.7 mSv), whereas in the remaining 38 (25%) patients retrospective scans were acquired (mean dose of 11.1±3.5 mSv). The overall mean radiation exposure was 4.9±4.0 mSv.

### Association of Calcified and Non-calcified Plaque to Biochemical Markers

**Figure 1:**
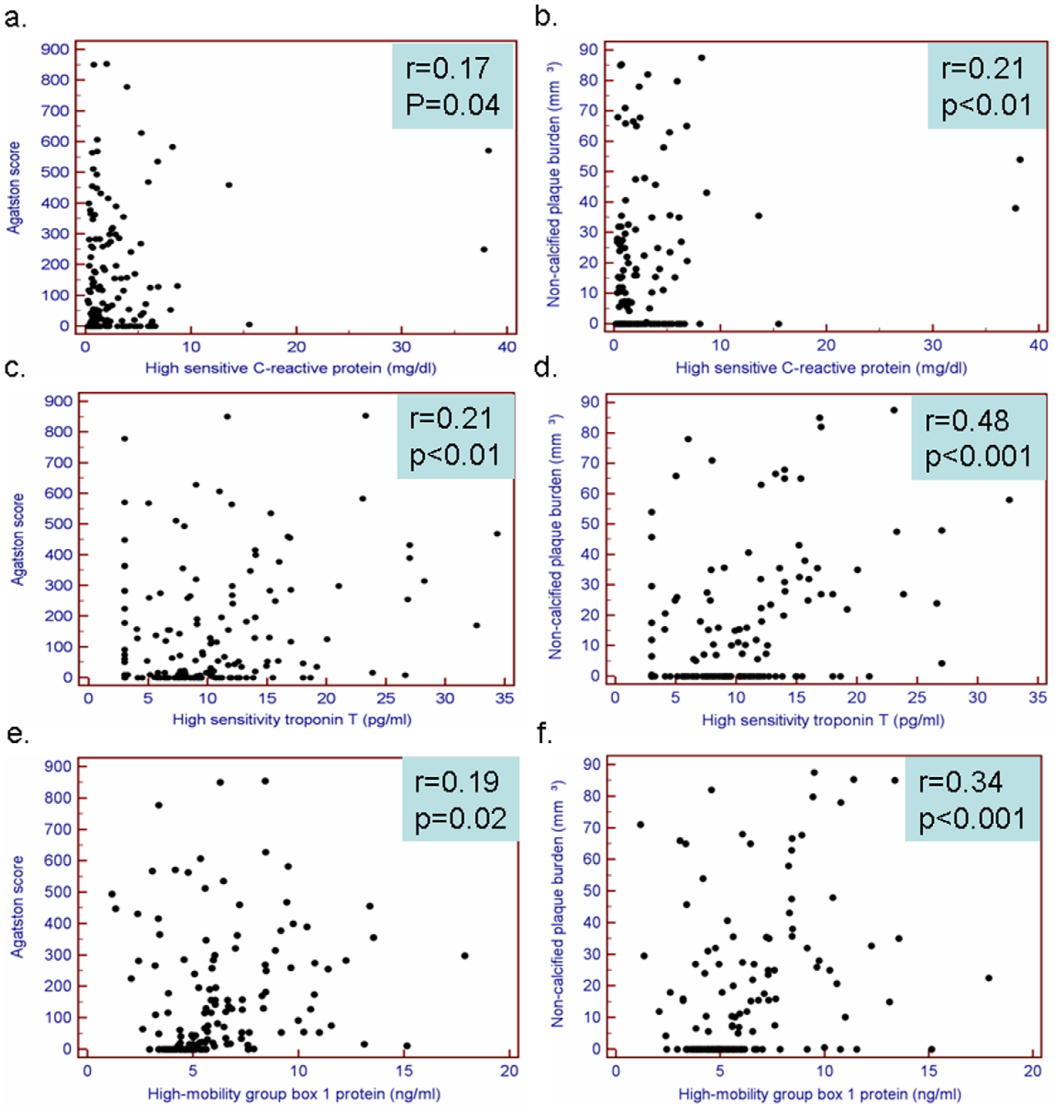
Weak correlations were observed between calcium scoring with hs-CRP, hs-TnT and HMGB1 (a, c and f). A weak correlation was also noted between hs-CRP and non-calcified plaque burden (b), while stronger correlations were observed between the latter with hs-TnT and HMGB1 (d and f).

**Figure 2:**
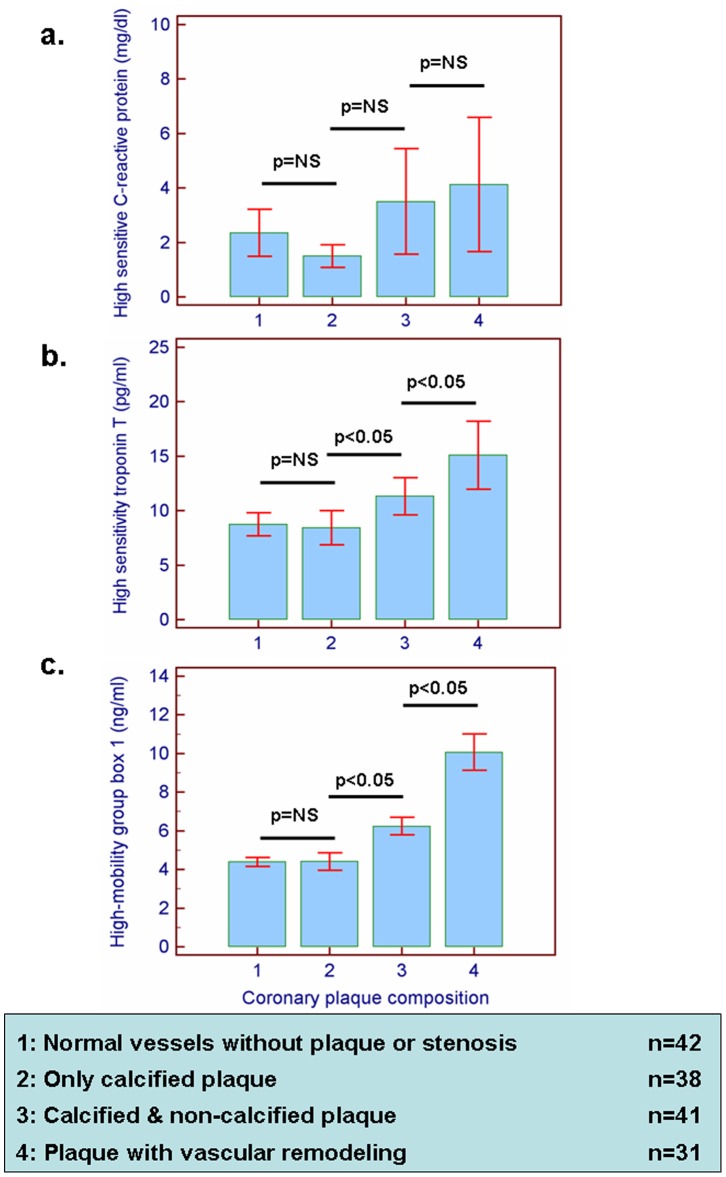
Classifying patients by plaque composition, a trend was observed for higher hs-CRP values in patients with non-calcified plaque without however, reaching statistical significance (a). HsTnT and HMGB1 values on the other hand, increased with increasing plaque presence and complexity, yielding higher values in patients with non-calcified plaque versus purely calcified or no plaques and the highest values in subjects with remodeled non-calcified plaque (b and c).

Weak correlations were observed between calcium scoring and hs-CRP, hs-TnT and HMGB1 (r = 0.17, r = 0.21 and 0.19, respectively, p<0.05 for all, Figure 1a, c and e). Furthermore, a weak correlation was noted between hs-CRP and non-calcified plaque burden (r = 0.21, p<0.01, Figure 1b), while stronger correlations were observed between the latter with hs-TnT and HMGB1 (r = 0.48 and r = 0.34, respectively, p<0.001 for both, Figure 1d and f). After classification of patients by plaque composition according to predefined criteria, a trend was observed for higher hs-CRP values in patients with non-calcified plaque (p = 0.09) (Figure 2a). HsTnT and HMGB1 values significantly increased with increasing plaque presence and complexity. The highest values were observed in subjects with remodeled plaque (Figure 2).

A correlation was observed between hs-TnT and HMGB1 (r = 0.26; p<0.01). No significant associations were noted between hs-TnT and hs-CRP or between hs-CRP and HMGB1 (data not shown).

### Prediction of Non-calcified Plaque Burden and of Plaque Composition by Biochemical Markers

Using univariate analysis the total number of atherogenic risk factors, hsCRP, hsTnT and HMGB1 were associated with non-calcified plaque. By multivariate logistic regression analysis HMGB1 and hsTnT were independent predictors for the presence of non-calcified plaque burden, whereas risk factors and hs-CRP were no longer significant (Table 2).

**Table 2:** Uni- and multivariable logistic regression analysis for the prediction plaque composition (no plaque and only calcified versus non-calcified plaque with or without vascular remodeling).

Variables	Coefficient	Odds Ratio	95% Confidence Interval (CI)	p-value
	**Univariate analysis**			
*Age(yrs.)*	0.03	1.03	0.99 to 1.07	0.06
*Male gender*	0.30	1.35	0.70 to 2.59	NS
*Arterial hypertension*	0.73	2.07	0.89 to 4.79	0.09
*Hyperlipidemia*	0.58	1.79	0.93 to 3.46	0.08
*Diabetes mellitus*	0.44	1.55	0.51 to 4.69	NS
*Positive family history*	0.47	1.60	0.84 to 3.05	NS
*Cigarette smoking*	0.24	1.27	0.66 to 2.43	NS
*Number of risk factors*	0.41	1.51	1.11 to 2.03	0.008
*Hs-CRP*	0.17	1.18	1.02 to 1.37	0.03
*Hs-TnT*	0.14	1.16	1.07 to 1.24	0.0001
*Hmbg1*	1.23	3.56	2.29 to 5.54	<0.0001
	**Multivariable analysis**			
*Age(yrs.)*	0.008	1.0	0.95 to 1.06	NS
*Number of risk factors*	0.48	1.6	0.98 to 2.65	0.06
*Hs-CRP*	0.08	1.1	0.96 to 1,21	NS
*Hs-TnT*	0.19	1.2	1.07 to 1.37	<0.01
*Hmbg1*	1.44	4.2	2.43 to 7.31	<0.001

HR indicates risk ratios and CI the corresponding 95% confidence intervals.

Plaque characteristics by patient tertiles based on their hsTnT and HMBG1 values are presented in Table 3. Patients in the upper tertiles for both hsTnT and HMBG1 showed higher calcium scoring and non-calcified plaque burden versus those in the mid and lower tertiles. Furthermore, by combining both biomarkers, a very high negative predictive value for the presence of non-calcified and remodeled plaque (95% and 100% respectively) was noted in patients within the lower tertiles, which surpassed the negative predictive value of each biomarker separately. Similarly, patients in the upper tertiles for both biomarkers yielded high positive predictive values for non-calcified and remodeled plaques (96% and 77%, respectively), which also surpassed the positive predictive value of each biomarker separately.

**Table 3:** Calcium scoring, non-calcified plaque volume and plaque composition by hsTnT and HMBG1 tertiles.

	*Calcium scoring (Agatston Units)*	*Non-clacified plaque burden (mm^3^)*	*Presence of non-calcified plaque*	*Presence of remodeled plaque*
	**HsTnT tertiles**			
*HsTnT lower tertile (3.0–7.9 pg/ml) N = 50*	134±185	10.0±17.9	37%	17%
*HsTnT mid tertile (8.0–11.7 pg/ml) N = 51*	107±186	6.5±13.4	33%	10%
*HsTnT upper tertile (11.8–34.4 pg/ml) N = 51*	206±203*	31.5±27.6*	72%	42%
	**HMBG1 tertiles**			
*HMBG1 lower tertile (1.2–4.7 ng/ml) N = 51*	114±194	11.5±21.4	12%	2%
*HMBG1 mid tertile (4.8–6.1 ng/ml) N = 50*	101±150	6.5±13.7	38%	2%
*HMBG1 upper tertile (6.2–17.9 ng/ml) N = 51*	228±209*	29.3±26.1*	92%	61%
	**Lower and upper tertiles for HMBG1 & HsTnT**			
*HMBG1 & HsTnT lower tertiles N = 20*	171±242	11.0±20.6	**5%**	**0%**
*HMBG1 & HsTnT upper tertiles N = 26*	271±211†	43.7±25.1†	**96%**	**77%**

* p<0.05 versus mid and lower tertiles;

† p<0.05 versus lower tertiles.

### Prediction of Clinical Outcomes by Plaque Composition and Biochemical Markers

During a mean follow-up duration of 3.2±0.9 years 19 MACE were recorded, including 6 deaths, 2 myocardial infarctions and 11 revascularization procedures in 147 patients. Five patients were lost during follow-up (3%). Logistic regression analysis demonstrated that non-calcified plaque burden, hs-TnT and HMBG1 were significant predictors of clinical outcome (Table 4).

**Table 4:** Non-calcified plaque burden and biochemical markers for the prediction of clinical outcomes (combined endpoint for death, myocardial infarction and coronary revascularization).

Variables	Coefficient	Odds Ratio	95% Confidence Interval (CI)	p-value
*Age(yrs.)*	0.05	1.05	0.99 to 1.10	0.06
*Number of risk factors*	0.31	1.37	0.90 to 2.09	NS
*Non-calcified plaque burden*	0.02	1.02	1.00 to 1.04	<0.05
*Hs-CRP*	0.06	1.06	0.98 to 1.14	NS
*Hs-TnT*	0.08	1.08	1.00 to 1.16	<0.05
*Hmbg1*	0.22	1.25	1.07 to 1.46	<0.01

### Observer Variabilities

Inter- and intra-observer variability was 11% and 8% for the assessment of total plaque burden, and 1.8% and 1.3% for coronary calcium scoring, respectively. For differentiation by plaque composition inter- and intra-observer agreement rates were 90% (κ = 0.76) and 95% (κ = 0.88), respectively.

## Discussion

Our study demonstrates that besides hs-TnT, a well established marker of cardiovascular risk, HMGB1 is independently associated with non-calcified plaque burden in patients with clinically stable CAD. HMBG1 exhibits complementary value to hsTnT for the prediction of non-calcified plaque, which possibly implicates the involvement of different underlying pathophysiologic pathways in the release of the 2 biomarkers into the serum of such patients. Non-calcified plaque, hs-TnT and HMBG1 are all related to clinical outcome. HsCRP, on the other hand, a marker of systemic low grade inflammation is not independently associated with non-calcified plaque burden and with plaque composition.

Several lines of evidence suggest that inflammation has a key role in the pathogenesis of atherosclerosis, plaque remodeling and transduction of the effects governed by conventional CAD risk factors [3], [4]. In this regard, markers of vascular inflammation and metabolic risk factors have been previously identified as predictors of adverse clinical outcomes in patients with CAD [4]. However, since the underlying pathophysiologic mechanisms are not yet fully understood, to date, such marker-orientated therapeutic strategies are not implemented in the clinical realm [15].

Experimental data point to the early involvement of monocytes and macrophages during atherogenesis development [16]. HMGB1, an ubiquitous nuclear protein, constitutively expressed in quiescent cells [17], has been detected within atherosclerotic plaques, being released by lesional macrophages, necrotic cells and foam cells [9], [18]–[20]. In further studies, HMBG1 has been reported as a potent mediator of tissue remodeling in experimental atherosclerosis and in diabetes [19], [21]–[23]. In addition to these experimental proofs, Yan-et al recently elegantly demonstrated that HMGB1 levels are associated with angiographically significant CAD both in nondiabetic and in type 2 diabetic patients in a large patient cohort (n = 512) [24], whereas we previously showed that a close association is present between HMGB1 and infarct size in patients with acute coronary syndromes [9], [10].

In the present study, we observed significant associations between HMGB1 and coronary plaque composition. HMGB1 was higher in patients with non-calcified plaque and the highest in patients with more complex ‘remodeled’ lesions. This is in line with previous experimental data, where high expression of HMGB1 could be demonstrated especially in cells driving the process of plaque remodeling such as monocytes, macrophages, dendritic and smooth muscle cells [20], [23] and with recent clinical data [24]. Considering both pathophysiologic mechanisms, i.e. inflammation or cellular stress for HMBG1 and necrosis for hsTnT it is not surprising that complementary value was observed for the two biomarkers, in terms of prediction of non-calcified and remodeled plaque. Thus, patients with both increased hsTnT and HMBG1 yielded high rates of vulnerable, non- calcified and remodeled plaque (96% and 77%, respectively) compared to patients with only increased either HsTnT or HMBG1. Similarly, a very high negative predictive value for the presence of non-calcified and remodeled plaque (95% and 100% respectively) was noted in patients within the lower tertiles for both biomarkers, which surpassed the negative predictive value of each biomarker separately (Table 3). In agreement to previous studies both non-calcified plaque and hs-TnT were related to clinical outcome [6], [7], [25], whereas the predictive value of HMBG1 levels was found to be similar to that of cardiac hs-TnT.

**Figure 3:**
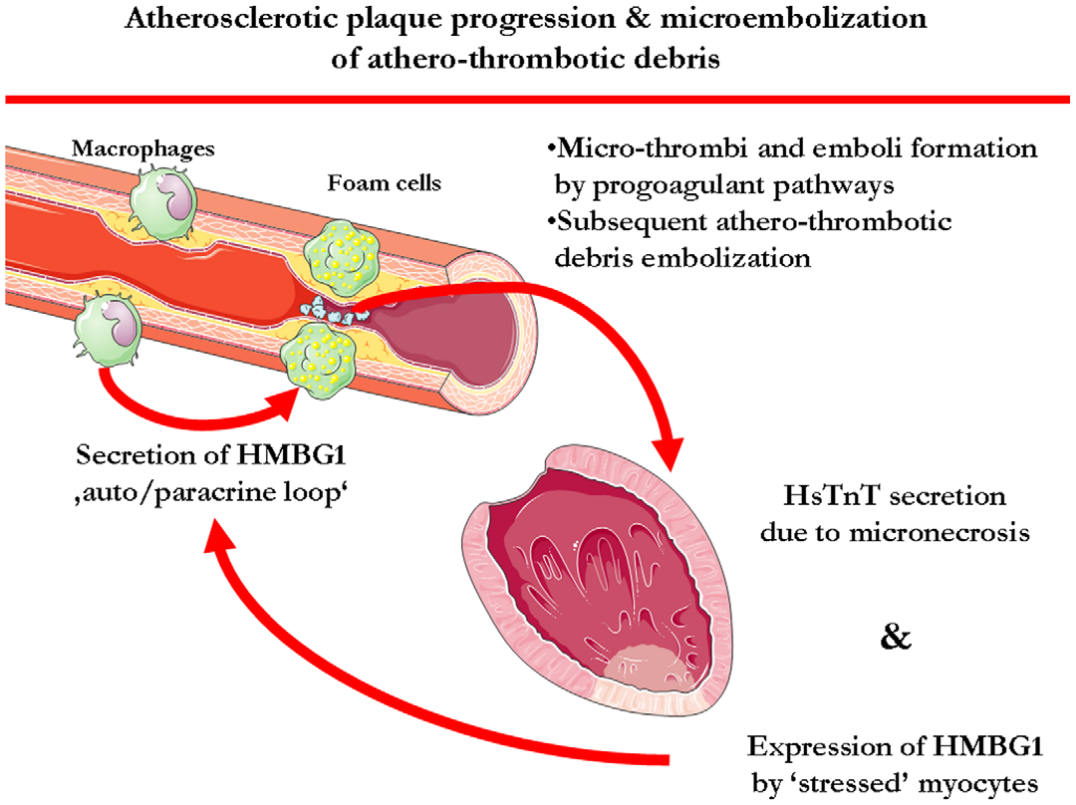
Expression of HMGB1 in lesional macrophages could promote vascular inflammation and ultimately cause plaque remodeling. Such remodeled, rupture-prone plaques may then cause chronic sub-clinical embolization of athero-thrombotic debris, which then results (i) in myocardial micro-necrosis, as reflected by the concomitantly increased hs-TnT values in the same patient subgroups and (ii) in further release of HMGB1 by ‘stressed’ cardiomyocytes. Increased HMBG1 expression would then elicit further pro-inflammatory response, again contributing to vascular remodeling processing, thus possibly being part of a vicious circle, which encompasses both chronic plaque inflammation and myocardial micronecrosis.

Although, our data *cannot provide an explanation of causality* it is conceivable, that expression of HMGB1 in lesional macrophages could promote vascular inflammation and vascular remodeling, as detected with CCTA images. Such remodeled, rupture-prone plaques may cause chronic sub-clinical embolization of athero-thrombotic debris, resulting in myocardial micro-necrosis and further release of the alarmin HMGB1 by ‘stressed’ cardiomyocytes[26]. Increased HMBG1 expression may then elicit further pro-inflammatory and pro-coagulant response[27], possibly being part of a vicious circle, which encompasses both chronic plaque inflammation, atherothrombosis and myocardial micronecrosis[28]. A schematic illustration of such interactions between the vascular and the myocardial bed can be appreciated in Figure 3.

In previous studies we and others reported a close relation between hs-TnT and plaque composition, while an independent association with hs-CRP could not be established [11], [12]. Such increased hs-TnT levels in our patients are to be interpreted as a consequence of irreversible myocyte death, caused by repetitive clinically silent plaque ruptures and micro-embolization into the terminal vasculature, which may precede the clinical manifestation of infarction or sudden cardiac death [29]. In this regard, earlier angioscopy studies demonstrated that plaque rupture and thrombosis are present in a considerable amount of patients with clinically stable CAD and may be a potential source of chronic troponin leakage due to micro-infarctions in this setting [29]–[31]. In the recently published PEACE study, hsTnT levels were independently associated with the incidence of cardiovascular death and heart failure in patients with stable coronary artery disease [25]. This supports the notion that hsTnT may serve as a biomarker for such ‘vulnerable’ coronary lesions even in presumably stable CAD and may together with HMBG1 present valuable therapeutic targets in such patients. In this regard, recent experimental data suggest that monoclonal anti-HMGB1 neutralizing antibodies reduce the development of atherosclerosis in apolipoprotein e-deficient mice [32]. HsCRP on the other hand, a marker of systemic low grade inflammation on the other hand, was not independently associated with plaque composition or with clinical outcome. Despite previous experimental evidence that, dissociation of the pentameric CRP may contribute to inflammation and atherosclerosis via pro-inflammatory effects of monomeric CRP [33], our current findings are in agreement with recent human genetic data indicating that CRP itself is highly unlikely to be even a modest causal factor in CAD [34] and with the general notion of limited value of this marker for individual clinical risk profiling [35], [36].

Of course, it should be acknowledged that increased hs-TnT and HMBG1 levels may reflect increased demand ischemia, myocardial strain because of volume or pressure overload, and impaired cell membrane integrity due to systemic inflammatory response or apoptosis (reviewed in [17], [37], [38]). Although in our cohort clinical conditions such as sepsis, myocarditis, chemotherapy and congestive heart failure were not present clinically, and preserved ejection fraction without regional wall motion abnormalities was documented by echocardiography in all patients, the contribution of such variables to hsTnT and HMBG1 elevation cannot be completely excluded.

From a technical point of view CCTA represents a non-invasive imaging technique, which on top of the assessment of lumen narrowing allows the evaluation of atherosclerotic plaque composition. In this regard, recent studies demonstrated its prognostic value in patients with stable CAD [6], underscoring the role of positively remodeled coronaries in areas of non-calcified plaque for the induction of future cardiac events [7], [8], [39], [40]. Of course the foremost limitation of CCTA is its association with radiation exposure, which limits its serial applicability in interventional therapeutic studies. However, recent radiation dose reduction strategies, which currently allow for low-dose CCTA with ∼1.0 mSv [41] in most patients, may be able to reduce this limitation in future trials.

Further limitations include the relatively small number of patients, the cross sectional nature of our study and the rather weak associations between HMBG1 and plaque composition. In addition, the influence of other pro-inflammatory biochemical markers such as soluble adhesion molecules, myeloperoxidase, atrix metalloproteinases and sCD40 were not investigated in this context, which is a limitation. Thus, larger scale biomarker studies are now warranted in order to assess the pathophysiological relevance of the findings reported herein. In addition, the ability of CTA for the differentiation of lipid-rich from fibrous plaque content is limited, due to substantial overlap of the corresponding attenuation values[42]. In this context intravascular ultrasound measurements or spectral CCTA[43] may be helpful to further verify the composition of atherosclerotic plaque in future studies.

### Conclusions

Our study demonstrates for the first time an association between CTA plaque characteristics and HMGB1 expression in patients with stable coronary artery disease. Although an explanation of causality cannot be supported by the present data, it is conceivable that a continuous pathophysiologic interaction between the coronary and myocardial bed, which encompasses HMBG1 secretion, plaque progression, embolization of athero-thrombotic debris and myocardial cell micro-necrosis with consecutive hsTnT leakage or myocyte apoptosis followed by further HMBG1 secretion, may explain our findings. Future trials are now warranted to test if such biomarkers can be used as therapeutic targets in patients with stable CAD.
