# Correction: HMGB1 Is Associated with Atherosclerotic Plaque Composition and Burden in Patients with Stable Coronary Artery Disease

**DOI:** 10.1371/journal.pone.0099246

**Published:** 2014-05-28

**Authors:** The PLOS ONE Staff

Dr. Bjoern Maack is not included in the author byline. He should be listed as the third author, and his affiliation is 1: Department of Cardiology, University of Heidelberg, Heidelberg, Germany.

The contributions of this author are as follows: performed the experiments, contributed reagents/materials/analysis/tools, wrote the manuscript.

The correct citation is: Andrassy M, Volz HC, Maack B, Schuessler A, Gitsioudis G, Hofmann N, et al. (2012) HMGB1 Is Associated with Atherosclerotic Plaque Composition and Burden in Patients with Stable Coronary Artery Disease. PLoS ONE 7(12): e52081. doi:10.1371/journal.pone.0052081
